# Multigenerational SOCS1 insufficiency: Implications for the pathogenesis of allergic and autoimmune disease

**DOI:** 10.70962/jhi.20250126

**Published:** 2026-04-09

**Authors:** Cliodhna Murray, Michael J. Harrison, Timothy R. Leahy, Caríosa Lee-Brennan

**Affiliations:** 1 Immunology Department, Galway University Hospital, Galway, Ireland; 2 Department of Respiratory Medicine, Galway University Hospital, Galway, Ireland; 3 Paediatric Immunology, Children’s Health Ireland, Dublin, Ireland; 4 Trinity College, University of Dublin, Dublin, Ireland

## Abstract

This letter highlights diagnostic importance of considering monogenic immune dysregulation in complex allergic and autoimmune diseases. Additionally, the report expands on the clinical spectrum of suppressor of cytokine signaling 1 insufficiency (SOCS1 insufficiency) by identifying severe, treatment-refractory eosinophilic asthma as a novel primary manifestation.

Suppressor of cytokine signaling 1 insufficiency (SOCS1 insufficiency) was first described in humans in 2020 as a rare monogenic disorder of immune dysregulation. SOCS1 insufficiency is characterized by a spectrum of autoimmunity, autoinflammation, and increased susceptibility to infection. To date, over 119 individuals with SOCS1 insufficiency have been reported worldwide with marked clinical heterogeneity, including among individuals with the same pathogenic variant (1).

SOCS1 is a key negative regulator of the Janus kinase/signal transducer and activator of transcription (JAK/STAT) pathway. Following cytokine stimulation of type 2 cytokine receptors, SOCS1 acts as a classical feedback inhibitor by binding directly to activated JAKs, preventing further phosphorylation of STATs and thereby modulating downstream transcription of cytokine responsive genes. Loss of SOCS1 leads to unregulated activation of the JAK/STAT signaling pathway and exaggerated cytokine responses, leading to the wide array of immune-mediated manifestations observed in affected individuals (2).

Herein, we describe a multigenerational family in which four individuals carry an identical heterozygous pathogenic autosomal-dominant SOCS1 exon 2 Ala9Profs*76 variant, yet display diverse clinical phenotypes (Fig 1 a). This deletion leads to a frameshift and a premature stop codon. Although these cases were previously published in *Nature Communications*, case 4 was originally reported as an asymptomatic carrier (2). All four family members were included in the recent international registry-based cohort analysis of SOCS1 insufficiency, in which they contributed to the registry dataset without detailed longitudinal phenotyping (1).

**Figure 1.**
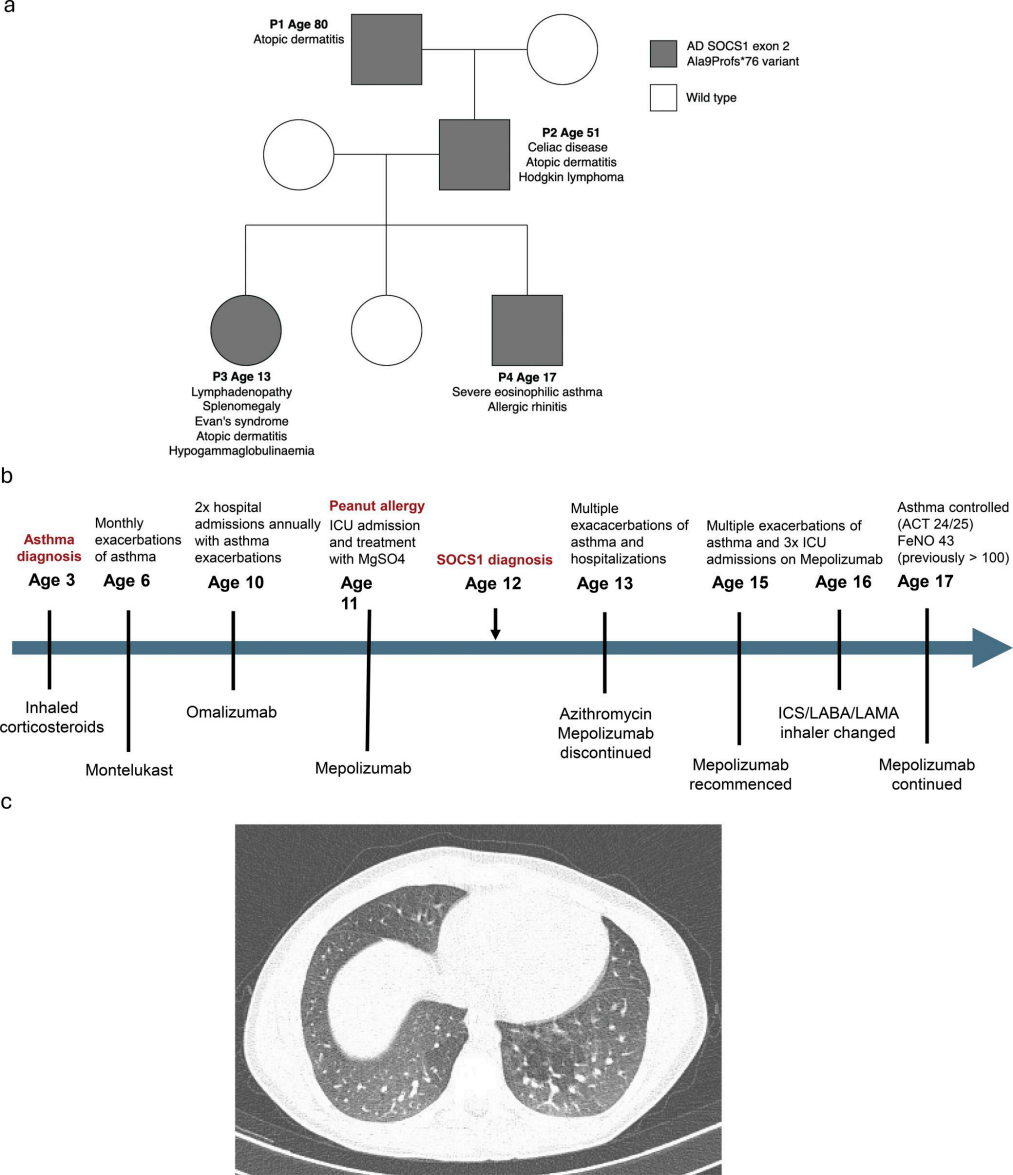
**Clinical manifestations of the affected family.** **(a)** Pedigree chart illustrating phenotypic manifestations within the kindred. **(b)** Timeline of disease development and treatment response in case 4. **(c)** Computer tomography of the thorax in case 4 demonstrating diffuse bronchial wall thickening and areas of air trapping in the medial basal segments of both lower lobes, consistent with airway inflammation. ICU, Intensive Care Unit; ACT, Asthma Control Test; ICS, Inhaled Corticosteroid; LABA, Long-Acting Beta2-Agonist; LAMA, Long-Acting Muscarinic Antagonist.

This report adds to the growing phenotypic spectrum of SOCS1 insufficiency and includes the first documented case of early-onset, severe, treatment-refractory eosinophilic asthma as a primary presenting feature.

Case 1 (paternal grandfather, age 80): The grandfather presented in infancy with severe eczema. Otherwise, he remains clinically unaffected into late adulthood, with no further history of autoimmune, atopic, or infectious complications.

Case 2 (father, age 51): The father presented at 2 years of age with early-onset coeliac disease and eczema. At 33, he was diagnosed with Hodgkin lymphoma, which was successfully treated with ABVD (doxorubicin, bleomycin, vinblastine, and dacarbazine) chemotherapy and consolidative radiotherapy. He has not developed significant infections or other autoimmune features since then.

Case 3 (daughter, age 13): The daughter presented at 3 years of age with persistent cervical lymphadenopathy, splenomegaly, Evans syndrome (autoimmune hemolytic anemia and immune thrombocytopenia), eczema, and mild hypogammaglobulinemia. Genetic testing identified the SOCS1 variant, which subsequently led to screening of her family members.

Case 4 (son, age 17): The son developed severe eosinophilic asthma at age 2 with recurrent wheeze and a persistently raised blood eosinophil count up to 353 cells/μl. Throughout his childhood, he required multiple admissions to the intensive care unit for recurrent viral exacerbations, including influenza A, rhinovirus, and respiratory syncytial virus. His asthma was refractory to multiple lines of treatment, including inhaled corticosteroids, leukotriene receptor antagonists, anti-IgE (omalizumab), and anti-IL-5 (mepolizumab). He also has comorbid eczema and allergic rhinitis. His computed tomography (CT) thorax showed diffuse bronchial mural thickening and air trapping in the lower lobes bilaterally (Fig. 1 b). The course of his disease and treatment response are summarized in Fig. 1 c. The remainder of his family, including his mother and unaffected sister, are healthy with no history recurrent infections or autoimmunity.

These cases highlight several key features of SOCS1 insufficiency. Firstly, marked phenotypic variability and pleiotropy is noted, with clinical manifestations ranging from autoimmunity, malignancy, and allergic disease to asymptomatic carriage. This variability suggests involvement of additional genetic, epigenetic, or environmental modifiers. Future prospective studies will be required to determine whether distinct immunologic signatures underlie phenotypic variability among SOCS1-insufficient individuals.

To our knowledge, case 4 is the first reported case of eosinophilic asthma as the predominant manifestation of SOCS1 insufficiency. Pulmonary manifestations in reported individuals with SOCS1 insufficiency have included granulomatous lymphocytic interstitial lung disease, recurrent bacterial pneumonias, and allergic asthma (3). However, the severity and treatment resistance seen here warrants further attention. An important limitation of this report is the absence of contemporaneous immunologic profiling, including cytokine analyses and SOCS1 or downstream STAT signaling studies, particularly in case 4. However, given the retrospective nature of the clinical data and prior functional characterization of this pathogenic variant in affected family members (2), the present report focuses on delineating the clinical phenotype.

Asthma is recognized as a predominantly cytokine-driven disease. The majority of cytokines and alarmins implicated in asthma pathogenesis, including IL-4, IL-5, IL-13, and thymic stromal lymphopoietin (TSLP), signal via JAK-coupled receptors. In murine models, SOCS1 deficiency leads to increased airway hyperresponsiveness and eosinophilic inflammation. In this context, the absence of SOCS1 explains the pathogenesis underlying the severe asthma in case 4. The heterozygous pulmonary complications frequently associated with SOCS1 insufficiency point toward an important role of SOCS1 in maintaining lung immune homeostasis. Notably, SOCS1 expression in human bronchial epithelial cells has been shown to be inversely correlated with eosinophil infiltration and T helper 2 cell (Th2) cytokine production, suggesting that impaired SOCS1 function could exacerbate type 2–driven inflammation (4).

Therefore, this report broadens the clinical spectrum of SOCS1 insufficiency and reinforces the potential for monogenic disorders of immune dysregulation to present with common, yet unusually severe or refractory immune-mediated diseases. The significance of case 4 lies in reframing diagnostic considerations in severe, treatment-refractory asthma in clinical practice. Recognizing asthma and other complex or treatment-resistant immune conditions as a potential sentinel manifestation of SOCS1 insufficiency alters clinical management by prompting consideration of genetic testing, genetic counseling, monitoring for autoimmune or malignant complications, as well as evaluation for emerging targeted therapies. The early identification of monogenic diseases of immune dysregulation is important to inform precise clinical management and to enable preventative strategies across affected families.

Additionally, these cases provide further mechanistic insights into the pathophysiology of more prevalent diseases such as asthma and autoimmunity. The link between a defined monogenic immune regulatory defect and severe eosinophilic asthma supports growing interest in JAK/STAT pathway involvement in allergic inflammation (5). While JAK inhibitors are currently approved for certain autoimmune and atopic conditions, their utility in asthma, for example, via topical administration, remains investigational. Our observations suggest that patients with asthma driven by upstream signaling abnormalities, such as SOCS1 dysfunction, may benefit from targeted JAK inhibition.

## Consent statement

Written informed consent was obtained from all participants.
